# Unbiased Analysis Method for Measurement of Red Blood Cell Size and Velocity With Laser Scanning Microscopy

**DOI:** 10.3389/fnins.2019.00644

**Published:** 2019-06-28

**Authors:** Emmanuelle Chaigneau, Morgane Roche, Serge Charpak

**Affiliations:** INSERM U1128, Laboratory of Neurophysiology and New Microscopy, Université Paris Descartes, Paris, France

**Keywords:** laser scanning microscopy, microcirculation, multiphoton microscopy, blood flow, red blood cells velocity

## Abstract

Two-photon laser scanning microscopy is widely used to measure blood hemodynamics in brain blood vessels. Still, the algorithms used so far to extract red blood cell (RBC) size and velocity from line-scan acquisitions have ignored the extent to which scanning speed influences the measurements. Here, we used a theoretical approach that takes into account the velocity and direction of both scanning mirrors and RBCs during acquisition to provide an algorithm that measures the real RBC size and velocity. We validate our approach in brain vessels of anesthetized mice, and demonstrate that it corrects online measurement errors that can reach several 10s of percent as well as data previously acquired. To conclude, our analysis allows unbiased comparisons of blood hemodynamic parameters from brain capillaries and large vessels in control and pathological animal models.

## Introduction

Blood flow mapping is widely used to image brain activity in physiological or pathological conditions because of the tight coupling that links neuronal activation and functional hyperemia (Iadecola, 2017). RBCs undergo major deformation depending on blood flow dynamics within microvessels, in particular when they pass through capillaries that are smaller than their diameter (Suzuki et al., 1996; Zharov et al., 2006). This deformability is impaired in many pathological conditions as hereditary disorders (for example spherocytosis, elliptocytosis, ovalocytosis, and stomatocytosis), diabetes, hypercholesterolemia (reviewed by Tomaiuolo, 2014), or during infection by plasmodium (Tiburcio et al., 2012). At cellular resolution, RBCs flow, velocity, and shape are usually investigated with laser scanning microscopy, either with one-photon excitation and confocal detection for superficial vessels or transparent samples (Dirnagl et al., 1992; Villringer et al., 1994), or with multiphoton excitation for scattering tissue (Kleinfeld et al., 1998; Chaigneau et al., 2003; Shih et al., 2012; Kong et al., 2016). RBC velocity measurements are now commonly used to quantify changes of vascular dynamics in brain pathological models (Hutchinson et al., 2006; Schaffer et al., 2006; Shih et al., 2009; Autio et al., 2011; Kisler et al., 2017). Accurate measurement of RBC shape and velocity with laser scanning microscopy is therefore essential for accurate interpretation of data, comparison of data acquired in various experimental conditions or using other techniques.

RBC velocity is usually assessed with line-scans, i.e., repetitive scanning along a longitudinal portion of a vessel. Line-scans produce space-time (Xt) images on which each RBC leaves an oblique shadow from which the RBC velocity is calculated. Initial studies mostly focused at the smallest vessels, where RBC velocity ranges from zero to about 1 mm/s. RBC velocity was calculated using a simple velocity model: the ratio of the displacement of the RBC from one line to the successive one divided by the time between two lines. These two parameters were measured using image processing algorithms: either a distance measurement after binarization or SVD (Dirnagl et al., 1992; Villringer et al., 1994; Kleinfeld et al., 1998; Chaigneau et al., 2003). RBC velocity measurements were then extended to larger vessels, i.e., arterioles and venules, where velocity is higher and its quantification becomes a technical challenge in terms of image acquisition and analysis (Hutchinson et al., 2006; Kang et al., 2006; Shih et al., 2009; Drew et al., 2010; Rungta et al., 2018). For this reason, a variety of image processing algorithms have been proposed : line fitting (Zhang et al., 2005), Radon Transform (Drew et al., 2010; Chhatbar and Kara, 2013), LSPIV (Kim et al., 2012), and Fourier Transform (Autio et al., 2011). Nevertheless, no study questioned the velocity model and raised the point that time registration was approximate as it depends on the scanning velocity and direction.

Here, we performed a detailed theoretical analysis of the line-scan acquisition method, assessing the impact of scanning velocity and direction on RBC size and velocity. Based on this analysis, we provide new algorithms that measure accurately RBC size and velocity, avoiding quantification errors that reach up to 100% in high speed vessels. We then validate experimentally our theoretical approach by measuring blood velocity in small and large vessels of anesthetized mice.

## Materials and Methods

### TPLSM System

Imaging was performed using a custom-made acquisition system and LabVIEW software (National Instruments, Austin, TX, United States). Femtosecond laser pulses were delivered by a Ti:Sapphire laser (Mira, 120 fs pulse width, 76 MHz, Coherent, Santa Clara, CA, United States) and targeted on the sample with galvanometric mirrors (Cambridge Technology, Bedford, MA, United States) and relay lenses (f_1_ = 60 mm, f_2_ = 250 mm). Light intensity was controlled by an acoustic optical modulator (MT110B50-A1.5-IR-Hk, AA Optoelectronic, Orsay, France). The excitation light was focused through a LUMFLN 60XW NA 1.1 objective (Olympus, Tokyo, Japan). Epifluorescence light was collected with a FF705-Di01 dichroic (Semrock, Rochester, NY, United States) and photons were separated based on their wavelength with a DCXR 560 (Chroma, Bellows Falls, VT, United States) dichroic mirror. Green light was filtered with an E800SP2 and a GQ620/40nm (Chroma, Bellows Falls, VT, United States) and targeted onto a GaAsP photomultiplier tube (Hamamatsu, Japan). Signals at the output of the photomultiplier tube were amplified with custom-build electronics and sampled at 1.25 MHz. Typical scanning velocities and corresponding line-scan conditions are provided in the Supplementary Table.

### Experimental Model Surgery and Anesthesia

All animal care and experimentation were performed following the experimental protocols approved by the INSERM Animal Care and Use Committee (Protocol Nos. CEEA34.SC.122.12 and CEEA34.SC.123.12) and work was reported complying with the ARRIVE guidelines. 5 Adult Thy1-GCaMP6f (GP5.11) mice and 1 adult wild-type mouse from the Jackson laboratory were used, 3–12 months old, 25–35 g, both males and female, housed in 12-h light–dark cycle. Mice were chronically implanted with a cranial glass window over the olfactory bulb. The animal preparation was done similarly to previously described (Lyons et al., 2016). Mice were anesthetized with a bolus of ketamine-medetomidine (100 and 0.4 mg kg^-1^ body mass, respectively) injected IP. They breathed a mixture of air and supplementary oxygen (the final inhaled proportion of oxygen was ∼30%) and the body temperature was monitored with a rectal probe and maintained at ∼37°C by a feedback-controlled heating pad. A craniotomy was performed with a dental drill and care taken not to apply pressure to the bone and the area was regularly flushed with cool aqueous buffer solution to avoid damage or heating of the underlying tissue. A cover glass (100 μm thick) was used for the window and sealed in place with photopolymerizable dental cement, which was also used to form a head-cap in which a titanium head-bar was embedded. Mice were permitted to recover for at least 3 weeks before the experimental sessions began.

For experiments, mice were anesthetized either with ketamine-medetomidine (100 and 0.4 mg kg^-1^ body mass, respectively) injected IP or with isoflurane (3%, in air). Experiments were performed within 20–100 min following injection of anesthetic. Breathing rate (2–3 Hz, regular and rhythmic) was monitored with a pneumogram transducer (Biopac Systems, Goleta, CA, United States). Body temperature was maintained at ∼37°C using a heating pad.

### Measurements and Analysis of RBC Velocity

Fluorescein dextran (70 kDa, D1823, Invitrogen, Villebon-sur-Yvette, France) was administered intravenously by retro-orbital injections and excited at 890 nm. Line-scan recordings were performed for 5–30 s on capillaries and arterioles. An image of each vessel in which line-scans were performed was also collected. The vessel internal diameter was measured as the width of the fluorescent plasma in this image after processing with a 3x3 median filter.

### Quantification and Statistical Analysis

V_RBCapp_ was computed with the SVD method (Kleinfeld et al., 1998) over 1s long sections using LabVIEW (National Instruments, Austin, TX, United States). Measurements were then averaged over the whole recording.

For Figure 3B, data were pooled from six mice. The distribution of diameters, V_AA_ and V_AR_ for each V_scan_ is shown in Supplementary Figure 1. For each V_scan_, the relation between V_AA_ and V_AR_ was fit using IgorPro7 (Wavemetrics, Portland, ME, United States), creating a user-defined function corresponding to Eq. 9 and optimizing the only parameter, “V_scan._” Iterative curve-fitting was performed by minimizing the value of chi-square using the Levenberg–Marquardt algorithm and the confidence interval was calculated for V_scan_ at *p* < 0.05.

For Figure 3C, data was fit using IgorPro7 (Wavemetrics, Portland, ME, United States) using linear curve-fitting.

**FIGURE 1:**
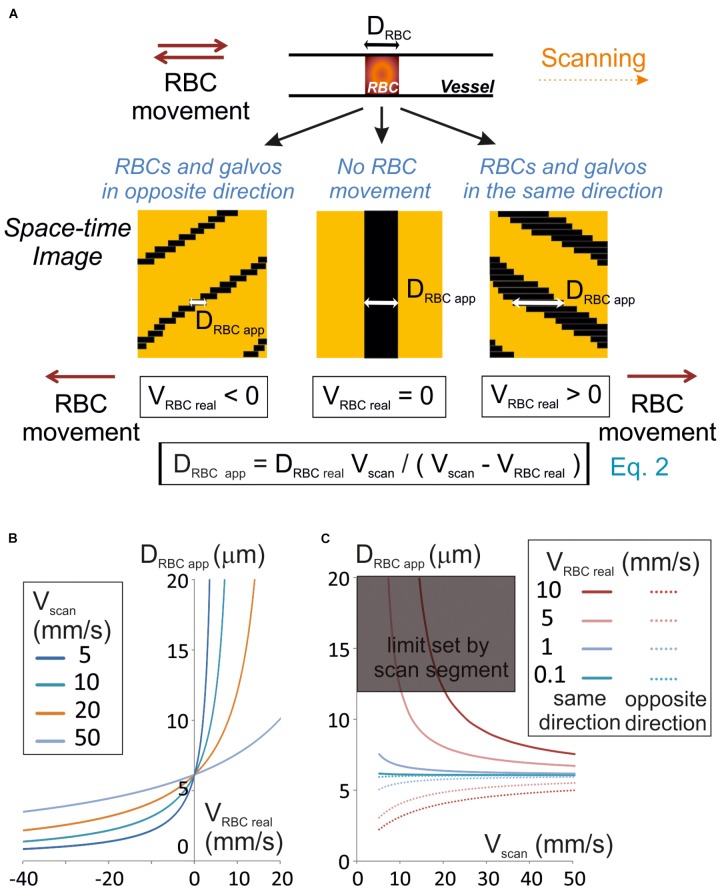
Unbiased measurement of RBCs size. **(A)** RBC apparent size (D_RBCapp_), depends on the scanning velocity (V_scan_) and direction. Scanning can be performed along or opposite to the RBC displacement (anterograde or retrograde scanning, respectively). Left panel: retrograde scanning; center panel: in the absence of RBC movement; right panel: anterograde scanning. A unique equation (Eq. 2) describes the relationship between D_RBCapp_, the real size of the RBC (D_RBCreal_), V_scan_ and the real velocity of the RBC (V_RBCreal_). **(B)** Anterograde scanning overestimates the RBC size whereas retrograde scanning underestimates the RBC size. The theoretical apparent size of a RBC (real size set to 6 μm diameter) was calculated from Eq. 2 and plot versus V_RBCreal_ for a range of V_scan_ typically used in standard two-photon microscopes. **(C)** Retrograde scanning allows accessing a larger range of RBC velocities. The theoretical apparent size of a RBC (real size set to 6 μm diameter) was calculated from Eq. 2 and plot versus V_scan_ for a range of V_RBCreal_. As the scan length sets a limit to D_RBCapp_, the range of scanning velocities that can be used to measure a given V_RBC_ covers a limited range of V_scan_ if scanning is performed in the same direction as blood flow, but the whole range of scanning speeds can be used if scanning is performed opposite to blood flow. The range of scanning velocities that can be used is further limited by the length of the scanned segment.

**FIGURE 2:**
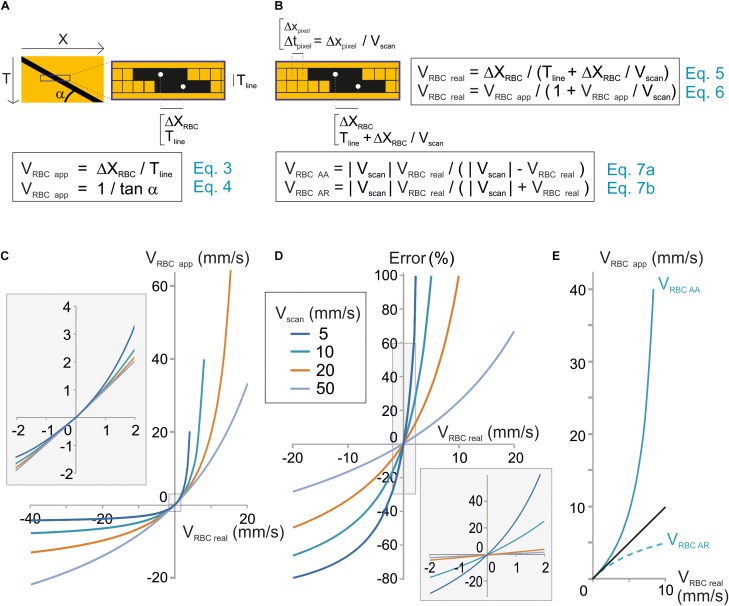
Unbiased measurement of RBC velocity. **(A)** Standard analysis used to calculate the apparent velocity of a RBC (V_RBCapp_) in space-time images. Scheme of the image parameters. V_RBCapp_ is the inverse of the slope of the shadow made by the RBC (angle α), which corresponds the ratio of the distance traveled by the RBC between two successive lines (ΔX_RBC_) divided by the time step between two lines (T_line_). **(B)** New analysis considering individual pixel time to calculate real velocity (V_RBCreal_). Top: scheme of the image parameters taking into account that each pixel within a line is acquired at a different time during scanning. The time step between two consecutive pixels (T_pixel_) depends on the pixel size (X_pixel_) and scan velocity (V_scan_). Therefore, the time to move by ΔX_mov_ from a line to the following one is actually T_line_ + ΔX_mov_/V_scan_. As a consequence, *V_RBCreal_* depends not only on T_line_ and ΔX_mov_ but also on V_scan_ as described by Eqs 5 and 6. These equations can be reformulated to calculate the anterograde apparent velocity of RBCs (V_RBCAA_) and the retrograde apparent velocity of RBCs (V_RBCAR_) as described by Eqs 7a,b. **(C)** V_RBCreal_ diverges from V_RBCapp_ when V_RBCreal_ ≥ 1 mm/s. V_RBCapp_ was calculated from Eq. 7 and plot versus V_RBCreal_ for a range of V_scan_. **(D)** The error made by using V_RBCapp_ instead of V_RBC_ can reach 10s of percent. The error made by using V_RBCapp_ instead of V_RBC_ was calculated as (V_RBCreal_ – V_RBCapp_)/V_RBCreal_ using Eq. 7 and plot versus exact V_RBC_ for a range V_scan_. Inset: enlargement of the plot initial part. **(E)** Anterograde scanning overestimates RBC velocity whereas retrograde scanning underestimates velocity. V_RBCAA_ and V_RBCAR_ were calculated from Eqs 7a,b and plot versus V_RBCreal_ for V_scan_ = 10 mm/s.

Simulations on Figure 1B,C, 2C–E, 3B were performed using Eqs 2, 7, 7a,b, and 9 with Microsoft Excel 2010. The error made by using V_RBCapp_ instead of V_RBCreal_ was calculated as (V_RBCreal_ – V_RBCapp_)/V_RBCreal_.

### Data and Code Availability Statement

Data and code used in the study are available upon direct request. This complies with the requirements of the funding institutes and with institutional ethics approval.

## Results

### Apparent Size of RBCs and Choice of Scanning Direction

Line-scans allow to estimate the RBC size along the vessel axis, that we will call RBC real size. So, we first investigated the relationship between the size of a RBC shadow, the RBC real size and the scanning velocity. If the RBC is still, neglecting the impact of the point spread function of the system, the size of the shadow equals the size of the RBC. The time for the scanning system to scan an RBC (T_RBC_) is the ratio of its real size (D_RBCreal_) by the scanning velocity (V_scan_) when the RBC is still (Figure 1A). For a moving RBC, the size of the shadow depends both on V_scan_ and the real RBC velocity (V_RBCreal_). Indeed the time needed by the scanning system to overtake the object depends on the relative speed between the scanning system and the object: V_scan_ – V_RBCreal_. Therefore, T_RBC_ is the ratio of D_RBCreal_ by V_scan_ – V_RBCreal_ (Figure 1A):

(1) $${\text{T}}_{\text{RBC}}={\text{D}}_{\text{RBCreal}}/({\text{V}}_{\text{scan}}-{\text{V}}_{\text{RBCreal}})$$

The size of the shadow, or apparent size of a RBC (D_RBC_ _app_), is thus given by the product of V_scan_, by T_RBC_:

(2) $${\text{D}}_{\text{RBCapp}}={\text{D}}_{\text{RBCreal}}{\text{V}}_{\text{scan}}{\text{/(V}}_{\text{scan}}-{\text{V}}_{\text{RBCreal}})$$

Therefore, if V_RBC_ _real_ < V_scan_, then D_RBC_ _app_ ≈ D_RBCreal_. Otherwise the apparent RBC size will be different from the RBC size. More precisely, D_RBC_ _app_ is larger than D_RBCreal_ when scanning is performed in the same direction as RBC flow (V_RBC_ > 0), and smaller than D_RBCreal_ when scanning is opposite to the flow (V_RBC_ < 0) (Figure 1A,B). If the scanning direction is the same as the RBC flow, for large V_RBC_ _real_, the scanning system will have difficulties to overtake RBCs, and D_RBC_ _app_ increases toward the infinite. The length of the scanned segment also sets a limit to the apparent size of RBCs. For all these reasons, scanning opposite to the direction of the RBC flow will allow accessing a larger range of RBC velocities than in the same direction as the RBC flow (Figure 1C).

As D_RBCreal_ varies depending on RBC orientation and vessel size, V_RBC_ cannot be estimated directly from Eq. 2. Thus measuring RBC speed requires visualizing the shadow at least on two successive lines of the space-time image obtained.

### Apparent RBC Velocity and True RBC Velocity

RBC velocity is usually calculated as the ratio of the displacement of the RBC from one line to the successive one ΔX_mov_ divided by T_line_, the time to acquire one line (Figure 2A). We will call this value the “apparent velocity” (V_RBCapp_):

(3) $${\text{V}}_{\text{RBCapp}}=\Delta {\text{X}}_{\text{mov}}/{\text{T}}_{\text{line}}$$

T_line_ is the sum of the time to scan the vessel segment, an eventual time to scan a region outside of the vessel and the time needed by the scanning system to “fly back” to the initial point of the scanned segment. The apparent velocity of a RBC corresponds to the inverse of the slope of the stripe it leaves on a space-time image (Figure 2A):

(4) $${\text{V}}_{\text{RBCapp}}=1/\tan(\alpha )$$

α being the angle made by the stripes and the horizontal line.

The mathematical model used in Eqs 3 and 4 would give the true RBC velocity if all pixels on a line were acquired at the same time, as with a camera. Nevertheless this is not the case with a scanning system, and that is why we call this velocity the “apparent velocity.” Indeed with a scanning system, pixels within each line are acquired at scanning speed. Therefore each pixel from each line of the space-time image is acquired at a different time. Two successive pixels are separated by T_pixel_ which is the ratio of the size of a pixel to the scanning system speed: X_pixel_/V_scan_ (Figure 2B). Therefore, the time used by the RBC to move by ΔX_mov_ from a line of the space-time image to the following one is actually T_line_ + ΔX_mov_/V_scan_. As a consequence, the mathematical model to calculate the real velocity of RBCs with a scanning system is:

(5) $${\text{V}}_{\text{RBCreal}}=\Delta {\text{X}}_{\text{mov}}{\text{/(T}}_{\text{line}}+\frac{\Delta {\text{X}}_{\text{mov}}}{{\text{V}}_{\text{scan}}})$$

This can also be expressed as:

(6) $${\text{V}}_{\text{RBCreal}}={\text{V}}_{\text{RBCapp}}\text{/(1}+\frac{{\text{V}}_{\text{RBCapp}}}{{\text{V}}_{\text{scan}}})$$

Therefore the real velocity of RBCs is different from their apparent velocity. Accurate measurement of RBC velocity requires correcting V_RBCapp_ by a factor depending on the scanning velocity.

Eq. 6 can be reformulated as:

(7) $${\text{V}}_{\text{RBCapp}}={\text{V}}_{\text{scan}}{\text{ V}}_{\text{RBCreal}}{\text{/(V}}_{\text{scan}}-{\text{V}}_{\text{RBCreal}})$$

Therefore, if V_RBCreal_ is much smaller than V_scan_, then V_RBCapp_ ≈ V_RBCreal_ (Figure 2C,D), otherwise V_RBCreal_ cannot usually be estimated accurately by V_RBCapp_ (Figure 2C,D).

Furthermore, V_RBC_ _app_ depends on the scanning direction. Absolute values of V_RBCapp_ for anterograde scanning, i.e., scanning in the same direction as flow (V_scan_ > 0), V_RBC_ _AA_ and retrograde scanning, i.e., scanning opposite to flow, V_RBC_ _AR_ will be defined as:

(7a) $${\text{V}}_{\text{RBCAA}}=|{\text{V}}_{\text{scan}}{\text{|V}}_{\text{RBCreal}}{\text{/(|V}}_{\text{scan}}|-{\text{V}}_{\text{RBCreal}})$$

(7b) $${\text{V}}_{\text{RBC AR}}=|{\text{V}}_{\text{scan}}{\text{|V}}_{\text{RBCreal}}{\text{/(|V}}_{\text{scan}}|+{\text{V}}_{\text{RBCreal}})$$

Anterograde scanning overestimates RBC velocity (V_RBCapp_ > V_RBCreal_ > 0), whereas retrograde scanning underestimates velocity (V_RBCreal_ > | V_RBCapp_|) (Figure 2E). Furthermore, for a given RBC real velocity and a given scanning velocity, the error made by using the apparent speed instead of the real RBC speed is larger with anterograde scanning than with retrograde scanning. As a consequence accurate measurement of RBC velocity requires taking into account both scanning velocity and direction.

Anterograde and retrograde apparent velocities are directly related by the following equation:

(8) $${\text{1/V}}_{\text{RBC AR}}-1/{\text{V}}_{\text{RBCAA}}=2/|{\text{V}}_{\text{scan}}|$$

Eq. 8 can be reformulated:

(9) $${\text{V}}_{\text{RBCAA}}=|{\text{V}}_{\text{scan}}|{\text{V}}_{\text{RBCAR}}/(|{\text{V}}_{\text{scan}}|-2{\text{V}}_{\text{RBCAR}})$$

### Experimental Validation

**FIGURE 3:**
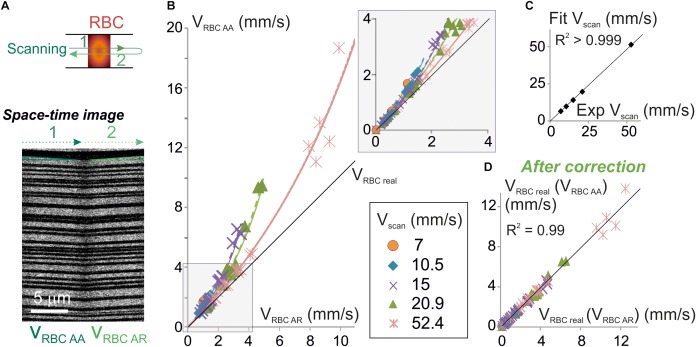
Experimental validation of the algorithm for unbiased measurement of RBC velocity. **(A)** Apparent velocities of RBCs were simultaneously measured with anterograde and retrograde scanning: each vessel was imaged using a line-scan pattern made of a single segment scanned in both directions (top scheme). Resulting space-time images (bottom picture) gave the anterograde apparent velocity of RBCs (V_RBCAA_) on their left half (anterograde scanning) and the retrograde apparent velocity of RBCs (V_RBCAR_) on their right half (retrograde scanning). The beginning and the end of each line was discarded from analysis to avoid periods of scanning acceleration and deceleration. **(B)** V_RBCAA_ is larger than V_RBCAR_ and their difference increases with V_scan_: experimental values of V_RBCAA_ (symbols) versus experimental values of V_RBCAR_ were plot_._ Each data point is a 2–10 s average value. Data was gathered from 5 to 31 bidirectional line-scans for each V_scan_ value (see details in Supplementary Figure 1 and see section “Materials and Methods”). Black line = unity line. The relation between V_RBCAA_ and V_RBCAR_ can be fit by Eq. 9: experimental values of V_RBCAA_ versus V_RBCAR_ were fit by Eq. 9: V_RBC_ _AA_ = | V_scan_| V_RBC_ _AR_ / (| V_scan_| - 2 V_RBC_ _AR_) (dashed colored lines) and compared to theoretical plots (full colored lines) for a variety of V_scan_. Inset: enlargement of the plot initial part. **(C)** Validation of the physical model to calculate V_RBCreal_: V_scan_ values obtained by fitting experimental data in **(B)** with Eq. 9 were compared to the experimental values of V_scan_. Their relationship was well fit by a line of slope 0.98 ± 0.01 (*R*^2^ > 0.999), confirming the validity of the theoretical model. **(D)** Validation of the velocity correction. V_RBCreal_ was computed from experimental values of V_RBCAA_ using Eq. 7a [giving V_RBCreal_ (V_RBCAA_)] and V_RBCAR_ using Eq. 7b [giving V_RBCreal_ (V_RBCAR_)]. The relation between V_RBCreal_ (V_RBCAA_) and V_RBCreal_ (V_RBCAR_) can be fit by a line of slope 1.00 ± 0.02 (*R*^2^ = 0.99), showing that both methods give the same values for V_RBCreal_ as predicted by our model.

Equation 9 can be used to test for the validity of our model as this equation only contains values that can be experimentally set or measured. Therefore, we have simultaneously measured V_RBC_ _AR_ and V_RBC_ _AA_ in a sample of 38 brain vessels from six mice whose diameters ranged from 2.3 to 17.3 μm (average 6.7 ± 4.5 μm, *n* = 38) (Figure 3A) using bidirectional line-scans over the same segment and performed a series of tests on this pool of experimental values. As predicted by our model, V_RBC_ _AA_ was always larger than V_RBC_ _AR_ (Figure 3B and Supplementary Figure 1). Furthermore, the difference between V_RBC_ _AR_ and V_RBC_ _AA_ increased both with V_scan_ and with V_RBC_ _AR_. The mathematical relation between experimental values of V_RBC_ _AR_ and V_RBC_ _AA_ was very close to the ones predicted by our model (Figure 3B) and very well fit using Eq. 9 (*p* < 0.05 for each fit corresponding to each V_scan_ value) (Figure 3B). V_scan_ values obtained by these fits had a very good match with real values of V_scan_ (Figure 3C) as their relation could be fit by a line of slope 0.96 ± 0.01 (*R*^2^ = 0.99), further confirming the validity of our mathematical model. V_RBCreal_ was then computed from experimental values of V_RBCAA_ using Eq. 7a [giving V_RBCreal_ (V_RBCAA_)] and from experimental values of V_RBCAR_ using Eq. 7b. [giving V_RBCreal_ (V_RBCAR_)] (Figure 3D). There was again a very good match between V_RBCreal_ (V_RBCAA_) and V_RBCreal_ (V_RBCAR_) (Figure 3D) as the mathematical relationship can be fit by a line of slope 1.02 ± 0.01 (*R*^2^ = 0.99). This completes the validation of our mathematical model to calculate V_RBCreal_ and the corrections to apply to measurements.

## Discussion

We have developed new algorithms to calculate RBC size and velocity with a line-scan acquisition method, that take into account the scanner movement. We have shown that measurements of RBC size and velocity can be erroneous if the scanning speed and direction are not considered. These errors can be avoided by using our algorithms, which provide unbiased models. Our algorithms cannot only be used for future measurements but also to correct for past measurements. Last, we have demonstrated the validity of our methodology by experimental measurements.

RBCs undergo severe deformations in capillaries in physiological and pathological conditions (Suzuki et al., 1996; Tomaiuolo, 2014). These deformations result in changes in their size along the vessel axis. Laser scanning microscopy is the method of choice to investigate these deformations in depth in living tissue. We have previously shown that RBC size increases with RBCs speed in capillaries where RBC speed is below 1 mm/s in the anesthetized rat (Chaigneau et al., 2003). Our new algorithm now allows extending such an analysis in conditions where RBCs speed is higher, and comparing pathological and physiological models.

RBC speed reaches 2–3 mm/s in brain capillaries, 10 mm/s in brain arterioles and more than 20–30 mm/s in larger pial vessels. Standard scanning systems provide scanning velocities from 5 to 20 mm/s (Kleinfeld et al., 1998; Chaigneau et al., 2003; Schaffer et al., 2006; Chhatbar and Kara, 2013; Kornfield and Newman, 2015; Dunn et al., 2018). For the later scanning velocity range, calculating RBC velocity without taking into account scanning speed and direction results in biased measurements. Resonant scanning mirrors, which provide scanning speed higher than 100 mm/s (Autio et al., 2011; Kim et al., 2012; Kisler et al., 2018), could overcome the problem but at the cost of restrictive conditions (the alignment of the vessel and scanning direction) and expensive upgrades. Our methodology allows to measure accurately RBCs velocity without any additional cost in all brain vessels. Having access to more and more animal models of brain pathology, numerous laboratories are now measuring resting blood velocity (Santisakultarm et al., 2014; Silva et al., 2014; Ishikawa et al., 2016; McConnell et al., 2016; Bragin et al., 2017; Liu et al., 2018). Accurate measurements are fundamental for comparison of data in a set of experimental conditions, within and in between laboratories. It will also allow obtaining the real RBC velocity in other body regions as laser scanning microscopy is also used in kidney (Kang et al., 2006; Dunn et al., 2018), retina (Kornfield and Newman, 2015), skin (Brown et al., 2001), hindlimb (Lasch et al., 2018), both in physiological and pathological condition. Furthermore, we have shown that retrograde scanning allows to sample higher RBC velocities that anterograde scanning for the same scanned segment. Therefore, retrograde scanning is advisable.

We have developed algorithms assuming that the scanning velocity was constant. Of course, scanning systems accelerate at the beginning of the scanned segment and decelerate at the end of the scanned segment, but most scanning systems are now able to stabilize their speed in less than 0.1 ms. Therefore, the scanning velocity is usually constant over most of the scanning. As acceleration and deceleration period distort RBCs shape and speed, the easiest approach to circumvent the problem is by discarding the corresponding sections of the spatiotemporal images. Nevertheless, in the eventuality of extremely short scanned segment, acceleration, and deceleration periods should be considered and algorithms could be adapted, providing that the exact kinetics of acceleration and deceleration of the scanning system are known.

Our RBC velocity algorithm can be used in combination to all kind of image analysis method (i.e., SVD (Kleinfeld et al., 1998), line fitting (Zhang et al., 2005), Radon transform (Drew et al., 2010; Chhatbar and Kara, 2013), LSPIV (Kim et al., 2012), and Fourier transform (Autio et al., 2011). Last but not least this correction can be used for diagonal line-scan (Kornfield and Newman, 2015).

Supplying oxygen, is one of the most critical roles of the brain vasculature (reviewed by Schmid et al., 2017). Local tissue oxygenation is directly linked to local blood flow and velocity. Under physiological conditions, in response to activation, both blood flow velocity and pO2 increases have been reported (Vazquez et al., 2010; Lecoq et al., 2011; Yaseen et al., 2011; Parpaleix et al., 2013). Prior the pO_2_ increase a brief and smaller decrease in pO_2_ has also been observed in capillaries and parenchymal regions close to capillaries (Lecoq et al., 2011; Parpaleix et al., 2013), reporting increased oxygen demand in the tissue. In the case of states of high energy expenditure, neurovascular coupling is essential for maintaining tissue oxygenation. It has been shown that, during sustained neuronal excitation, there was hardly any pO2 increase in tissue (Devor et al., 2011). Unbiased measurement of RBC velocity is fundamental for proper understanding of these mechanisms. Red blood cell morphology does not directly impact oxygen supply as the gas transport capacity of RBCs is not only determined by their morphology. Nevertheless, changes in RBCs morphology can alter their rheological properties, and as a consequence, modify oxygen supply. Under pathological conditions it has been shown that misshaping of RBCs due to sickle cell disease induces reduced hematocrit and, in some case increased, blood viscosity, which both contribute to reduced brain tissue oxygenation (Waltz et al., 2015).

## Conclusion

Accurate measurement of RBC velocity and size requires taking into account both scanning velocity and direction. Our analysis algorithms provide a costless way to measure accurately the real velocity of RBCs over a range of scanning velocities and to correct for past measurements.

## Data Availability

The datasets generated for this study are available on request to the corresponding author.

## Ethics Statement

All animal care and experimentation were performed following the experimental protocols approved by the INSERM Animal Care and Use Committee (Protocol Nos. CEEA34.SC.122.12 and CEEA34.SC.123.12) and work was reported complying with the ARRIVE guidelines.

## Author Contributions

EC initiated the project, developed the mathematical model and the software, and analyzed the data. EC and SC designed the experiments and wrote the manuscript. MR and SC performed the experiments. SC supervised the project.

## Conflict of Interest Statement

The authors declare that the research was conducted in the absence of any commercial or financial relationships that could be construed as a potential conflict of interest.

## Abbreviations

D_RBCapp,_: apparent size of a RBC
D_RBCreal,_: real size of an RBC
RBC: red blood cell
SVD: singular value decomposition
T_line_: the time to acquire one line
T_pixel,_: time step between two consecutive pixels
T_RBC,_: time to scan an RBC
V_RBCapp,_: apparent velocity of an RBC
V_RBCAA,_: absolute value of V_RBCapp_ for anterograde scanning
V_RBCAR,_: absolute value of V_RBCapp_ for retrograde scanning
V_RBCreal,_: real RBC velocity
V_scan,_: scanning velocity
X_pixel,_: pixel size
ΔX_mov,_: displacement of an RBC from one line of the line-scan to the next one
