# Supplementary material for: Unbiased Analysis Method for Measurement of Red Blood Cell Size and Velocity With Laser Scanning Microscopy
Source: Front Neurosci. 2019 Jun 28;13:644. doi: 10.3389/fnins.2019.00644 (PMC6610068; doi:10.3389/fnins.2019.00644)
Supplement: Supplementary file 1 [file Data_Sheet_1.docx]

**Supplementary information**

**Supplementary table**

| **Scanning speed (mm/s)** | | **Line length (μm)** | | | |
| --- | --- | --- | --- | --- | --- |
| **number of lines per second** | **Time / line (ms)** | **10** | **20** | **50** | **100** |
| **2000** | **0.5** | 30 | 60 | 150 | 300 |
| **1000** | **1** | 15 | 30 | 75 | 150 |
| **500** | **2** | 7.5 | 15 | 37.5 | 75 |
| **200** | **5** | 3 | 6 | 15 | 30 |
| **100** | **10** | 1.5 | 3 | 7.5 | 15 |

This table gives the average scanning speed for typical unidirectional line-scans conditions. The number of lines per second and time / line includes a “flyback time” during which the scanning system speed is twice the acquisition speed. Beware, this table is only indicative as it will depend on the exact scanning settings of the system, i.e. acceleration / deceleration period, unidirectional / bidirectional scanning and other system-dependent parameters.

**Supplementary Figure 1**

**
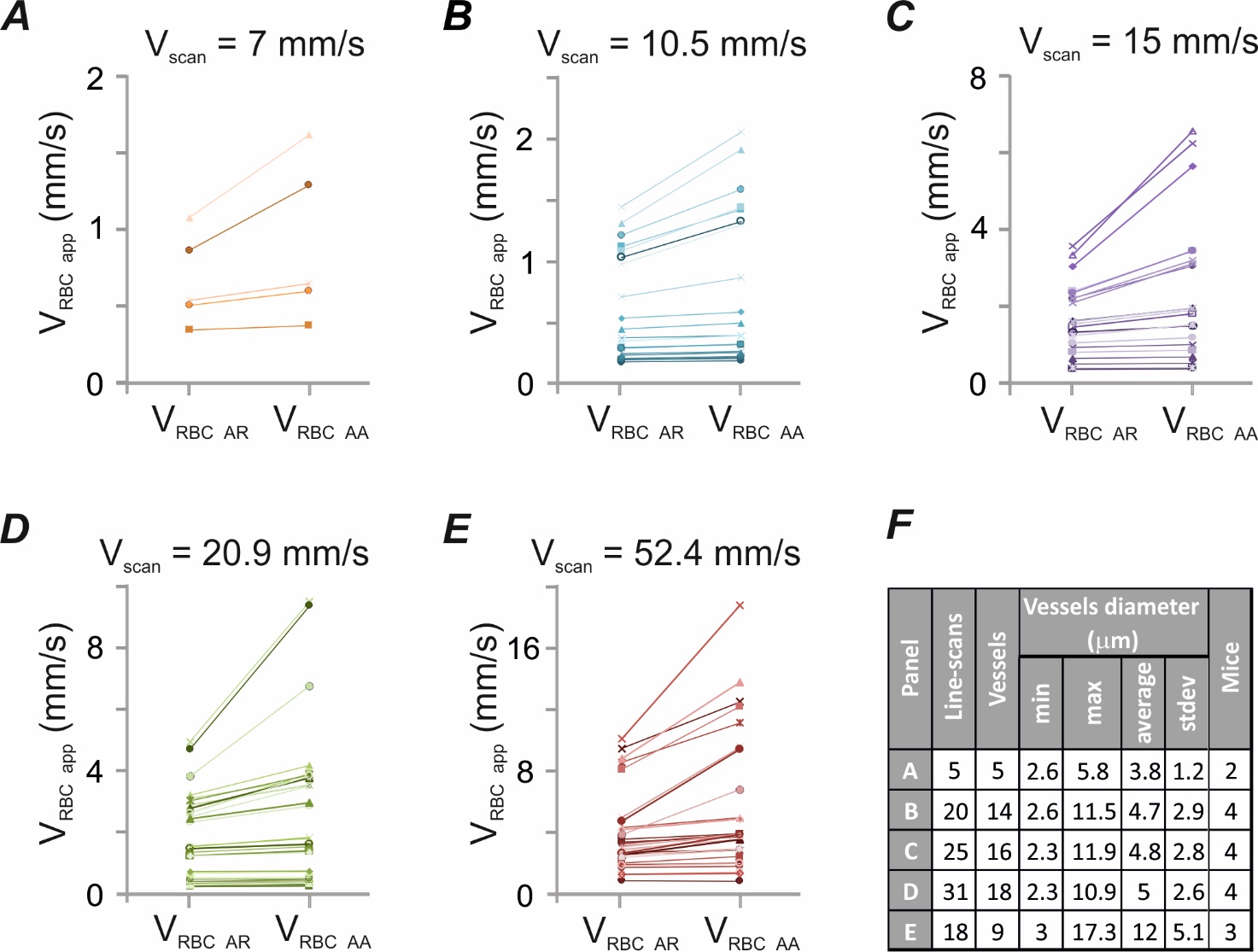
**

**Supplementary Figure 1:** A-E Experimental *V_AA_ and V_AR_ values for each V_scan_***.** F- Distribution of data.
